# Identifying longevity associated genes by integrating gene expression and curated annotations

**DOI:** 10.1371/journal.pcbi.1008429

**Published:** 2020-11-30

**Authors:** F. William Townes, Kareem Carr, Jeffrey W. Miller

**Affiliations:** 1 Department of Computer Science, Princeton University, Princeton, New Jersey, USA; 2 Department of Biostatistics, Harvard T.H. Chan School of Public Health, Boston, Massachusetts, USA; OvGU; Medical Faculty, GERMANY

## Abstract

Aging is a complex process with poorly understood genetic mechanisms. Recent studies have sought to classify genes as pro-longevity or anti-longevity using a variety of machine learning algorithms. However, it is not clear which types of features are best for optimizing classification performance and which algorithms are best suited to this task. Further, performance assessments based on held-out test data are lacking. We systematically compare five popular classification algorithms using gene ontology and gene expression datasets as features to predict the pro-longevity versus anti-longevity status of genes for two model organisms (*C. elegans* and *S. cerevisiae*) using the GenAge database as ground truth. We find that elastic net penalized logistic regression performs particularly well at this task. Using elastic net, we make novel predictions of pro- and anti-longevity genes that are not currently in the GenAge database.

## Author summary

Aging is a complex process with poorly understood genetic mechanisms. Recent studies have sought to classify genes as pro-longevity or anti-longevity using a variety of machine learning algorithms. However, it is not clear which types of features are best for optimizing classification performance and which algorithms are best suited to this task. Further, performance assessments based on held-out test data are lacking. We systematically compare five popular classification algorithms using gene ontology and gene expression datasets as features to predict the pro-longevity versus anti-longevity status of genes for two model organisms (nematode worms and yeast) using the GenAge database as ground truth. We find that elastic net penalized logistic regression performs particularly well at this task. Using elastic net, we make novel predictions of pro- and anti-longevity genes that are not currently in the GenAge database.

## Introduction

Identifying the genetic and molecular basis of aging is a longstanding goal in medical science [1, 2]. Advances in aging research have uncovered several common denominators of aging that are conserved across a wide range of organisms [3], and several drugs have been identified that have remarkable pro-longevity effects in model organisms [4]. However, much remains unknown about the biology of aging.

Many studies have investigated whether individual genes are pro-longevity or anti-longevity on a case-by-case basis [5]. Typically, an intervention such as a knockout/knockdown or overexpression is applied to a small number of genes in a model organism such as nematode worm (*Caenorhabditis elegans*) or yeast (*Saccharomyces cerevisiae*) followed by quantification of lifespan. A gene is considered *pro-longevity* if its expression is directly related to lifespan—for instance, if overexpression increases lifespan or underexpression decreases lifespan [6]. Conversely, a gene is considered *anti-longevity* if its expression is inversely related to lifespan. Meanwhile, many genes do not fall clearly into either category, for instance, a gene might have no discernable effect on lifespan. The GenAge database [6] contains a catalogue of putative pro- and anti-longevity genes based on current evidence.

Pro/anti-longevity genes can be identified by intervening on individual genes, but this is slow and expensive. Alternatively, a common technique is to randomly knock out or disrupt many genes in a population of organisms, screen for the longest living individuals, and then determine which genes were disrupted in these individuals. This screening technique can rapidly identify anti-longevity genes, but systematically identifying pro-longevity genes is less straightforward. Indeed, among the small number of genes annotated as having some impact on longevity in worms and yeast, there are considerably more anti-longevity genes than pro-longevity genes.

To prioritize which genes to investigate and speed up the discovery process, recent studies have sought to computationally predict the effect of gene interventions on aging, using annotations like Gene Ontology (GO) terms [7] as predictors. A survey of such efforts is provided by Fabris et al [8]. However, these recent studies suffer from several limitations. First, annotations like GO may be biased by the scope of the existing literature [9]. Second, it is difficult to compare results across studies since there is a lack of consistency in the choice of algorithms, feature sets, and predictive target/outcome. Finally, most recent studies do not report predictive performance on a held-out test dataset, leading to possible overestimation of performance.

We address these gaps by systematically assessing the performance of five popular machine learning algorithms on the task of predicting the pro- versus anti-longevity status of genes in *S. cerevisiae* and *C. elegans*. We use a consistent outcome in all comparisons based on GenAge annotations [6]. We compare the efficacy of GO terms versus gene expression profiles as feature sets for prediction. Further, we predict possible pro/anti-longevity genes that are not currently annotated in GenAge to suggest directions for future experimental studies.

## Results

### Data sources and algorithms

We compare the performance of five machine learning classification algorithms: elastic net penalized logistic regression (pglm) [10], support vector machine with radial basis function (svm) [11], gradient boosted trees (xgb) [12], naive Bayes (nb) [13], and k-nearest neighbors (knn) [14].

We define the outcome (that is, the target of prediction) to be the pro- versus anti-longevity annotation of individual genes from GenAge. After data cleaning, we identified 398 yeast genes and 848 worm genes with unambiguous annotations. Of these, the majority were labeled as anti-longevity (347 for yeast and 565 for worm). For validation and comparison, in yeast, we also consider replicative lifespan (RLS) outcome data for a comprehensive set of 4,698 single-gene deletions [15]; we refer to this as the McCormick dataset. In yeast, it is more common to use replicative lifespan rather than chronological lifespan to study aging.

As features for prediction, we consider using GO terms [7] and ARCHS4 gene expression profiles [16] for both yeast and worm. For yeast only, we also consider using the Deleteome dataset [17], which contains gene expression profiles for nearly 1500 single-gene deletions. For worm only, we also consider using the Worm Cell Atlas dataset [18], which contains gene expression profiles for around 50,000 cells. We write GXP to signify Deleteome and Worm Cell Atlas for yeast and worm, respectively. Altogether, we compare the performance of five feature sets for each species: (1) ARCHS4 alone, (2) GO alone, (3) GXP alone (Deleteome for yeast, Worm Cell Atlas for worm), (4) GO combined with ARCHS4, and (5) GO combined with GXP. Normalization, filtering, and other preprocessing steps are described in the Methods section.

To predict whether a particular gene *g* is pro- or anti-longevity, we construct features in the following manner. Each GO term is considered a separate binary feature taking a value of one if gene *g* is annotated to the term and zero otherwise. For the ARCHS4, Deleteome, and Worm Cell Atlas data each experimental condition (e.g., a perturbation or tissue sample) is considered a feature and its value is given by the expression of gene *g* under that condition. Note that this is the transpose of how gene expression data are usually investigated. However, by treating experimental conditions as features and genes as observations, this allows us to exploit arbitrary gene expression data for gene *g*, not just data from when *g* is perturbed.

### Comparative performance of algorithms and feature sets

To assess predictive performance, we use the following cross-validation scheme. For each of the two species, we split the GenAge-annotated genes into five cross-validation folds, and then for each combination of fold, algorithm, and feature set, we compute the area under the receiver-operator curve (AUC). Thus, in total, we compute 2 × 5 × 5 × 5 = 250 AUC values, 50 for each algorithm (S1 and S2 Figs).

**Fig 1:**
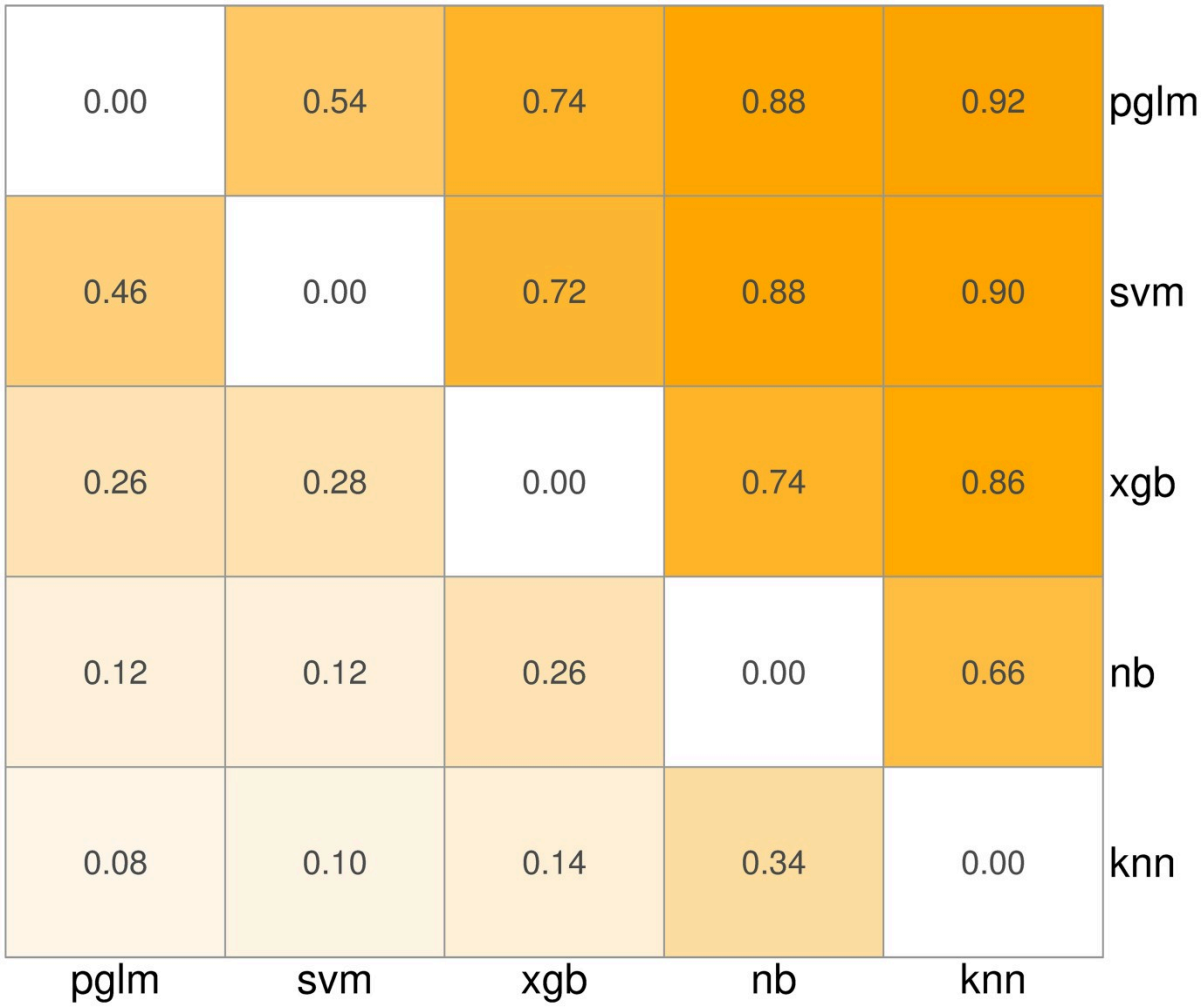
Ranking machine learning algorithms based on AUC. Numeric values indicate the fraction of times the row algorithm has higher classification performance than the column algorithm. pglm: elastic net penalized logistic regression, svm: support vector machine with radial basis function, xgb: gradient boosted trees, nb: naive Bayes, knn: k-nearest neighbors.

To summarize the relative performance of the five algorithms, Fig 1 shows how frequently algorithm *a* has higher AUC than algorithm *b* for each pair *a*, *b*. More precisely, for each pair of algorithms, Fig 1 shows the fraction of times algorithm *a* has higher AUC than algorithm *b* across the 50 combinations of species, fold, and feature set. The pglm and svm algorithms consistently outperform the others in terms of AUC. The ranking of algorithms is unchanged when compared using only yeast data. Using only worm data, svm slightly outperforms pglm (0.52 instead of 0.46 in Fig 1), and knn slightly outperforms nb (0.56 instead of 0.34 in Fig 1).

**Fig 2:**
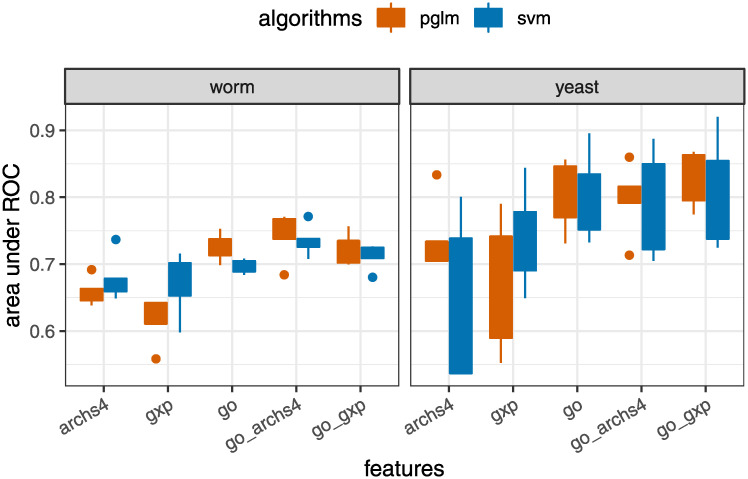
Combining gene expression (archs4, gxp) with gene ontology (GO) features yields improved classification performance in terms of AUC. pglm: elastic net penalized logistic regression, svm: support vector machine with radial basis function. An AUC value of 1 indicates perfect classification, whereas an AUC of 0.5 signifies performance no better than random.

To compare the relative performance of the five different feature sets, Fig 2 shows boxplots of the AUC values over the five cross-validation folds, stratified by species, algorithm, and feature set. For visual clarity, here we only show the results for pglm and svm (the two best algorithms); see S2 Fig for the other algorithms. Generally speaking, using GO terms yields better predictions than gene expression features alone (ARCHS4 or GXP). However, combining GO with gene expression (GO+ARCHS4 or GO+GXP) tends to increase AUC performance compared to GO alone.

Comparing gene expression feature sets, the ARCHS4 features give better performance than GXP (Worm Cell Atlas) for worms, but for yeast, GXP (Deleteome) is superior to ARCHS4. This could be simply due to the fact that the number of features in the worm ARCHS4 data is much larger than in the Worm Cell Atlas data. Alternatively, it could be due to the greater variation in experimental conditions across Deleteome features (which covers a comprehensive set of gene knockouts) compared to Worm Cell Atlas features (which consists of expression profiles of different cell types in normal worms).

**Fig 3:**
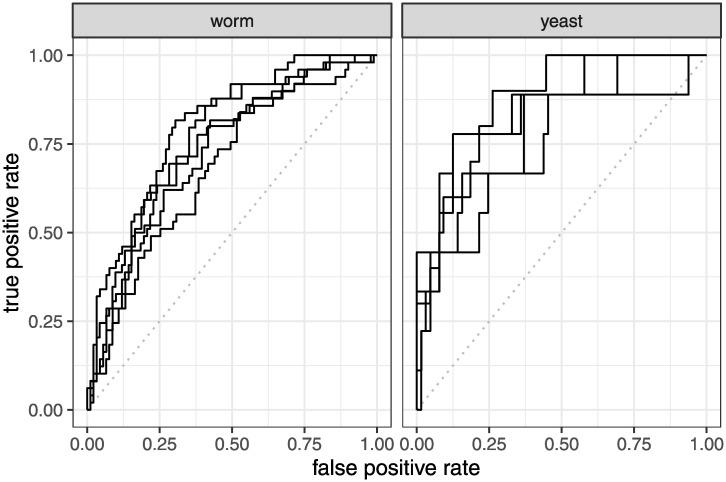
Receiver operator curves (ROC) for the best performing algorithm (pglm: Elastic net penalized logistic regression) with the best performing feature sets (GO+GXP for yeast and GO+ARCHS4 for worm). Each curve represents predictive performance on the held-out data from a single cross validation fold. The diagonal gray dotted line indicates the theoretical performance of an untrained random classifier as a baseline.

Overall, for worms, pglm with GO+ARCHS4 features yields the best performance, whereas for yeast, pglm with GO+GXP is best (Fig 3).

### Novel predictions of pro/anti-longevity genes

Given the encouraging performance of pglm for predicting pro/anti-longevity genes in GenAge, we applied the algorithm to make novel predictions of pro/anti-longevity genes in *C. elegans* (worm) and *S. cerevisiae* (yeast). To do this, for each species separately, we retrained a pglm model on the full GenAge database, using the combined GO terms plus ARCHS4 gene expression as features (see the Methods section for details on hyperparameter selection). Although for yeast the GO+GXP (Deleteome) features had slightly higher median predictive performance than GO+ARCHS4, we used the latter instead to maintain consistency across the two species. We then used the trained model to generate a predictive score for the pro/anti-longevity effect of each gene not in the GenAge database. Specifically, the predictive score is defined to be the probability that the gene is pro-longevity under the trained model. A score close to 1 indicates that the gene is predicted to be pro-longevity, whereas a score close to 0 indicates that the gene is predicted to be anti-longevity. An intermediate score indicates a gene with unclear pro- or anti-longevity status. Table 1 shows the unannotated genes with the highest confidence levels of being pro- and anti-longevity for worm and yeast, respectively. These genes do not significantly overlap with predictions from the pglm model trained using only GO terms as features (S2–S5 Tables, S3 Fig), suggesting that these predictions are not simply recapitulating the known biology represented in the GO terms. Complete lists of predictions for all genes are provided in S1 and S2 Data.

**Table 1:** Top pro-longevity and anti-longevity genes not in GenAge predicted using GO terms and ARCHS4 gene expression for worm and yeast with the pglm (GLM-Net) algorithm.

Species	Effect	Gene	Prob	ID	Description from ENSEMBL
worm	pro-longevity	CLEC-196	0.868	WBGene00009156	C-type LECtin
		F44E5.4	0.866	WBGene00009691	
		CEH-13	0.859	WBGene00000437	Homeobox protein ceh-13
		LPR-3	0.853	WBGene00012261	LiPocalin-Related protein
		HIL-7	0.845	WBGene00001858	HIstone H1 Like; Histone H1.Q
		W04A8.4	0.836	WBGene00012239	
		TTH-1	0.816	WBGene00006649	Thymosin beta
		GST-1	0.814	WBGene00001749	Glutathione S-transferase P
		F44E5.5	0.812	WBGene00009692	
		F20C5.6	0.807	WBGene00008971	
worm	anti-longevity	RPL-34	0.986	WBGene00004448	Ribosomal Protein, Large subunit
		MSP-59	0.985	WBGene00003452	Major sperm protein
		Y59E9AR.7	0.982	WBGene00022002	Major sperm protein
		RPL-39	0.982	WBGene00004453	60S ribosomal protein L39
		MSP-57	0.981	WBGene00003450	Major sperm protein
		MSP-81	0.981	WBGene00003467	Major sperm protein
		MSP-113	0.979	WBGene00003468	Major sperm protein
		MSP-19	0.978	WBGene00003426	Major sperm protein
		NLP-27	0.977	WBGene00003765	Neuropeptide-Like Protein
		RPL-11.1	0.977	WBGene00004422	60S ribosomal protein L11-1
yeast	pro-longevity	ACS1	0.882	YAL054C	Acetyl-coA synthetase isoform
		UBC5	0.863	YDR059C	Ubiquitin-conjugating enzyme
		ETR1	0.824	YBR026C	2-enoyl thioester reductase
		UBI4	0.779	YLL039C	Ubiquitin
		PDI1	0.72	YCL043C	Protein disulfide isomerase
		PRE3	0.713	YJL001W	Beta 1 subunit of the 20S proteasome
		POR1	0.705	YNL055C	Mitochondrial porin (voltage-dependent anion channel)
		PRE7	0.701	YBL041W	Beta 6 subunit of the 20S proteasome
		HSP12	0.698	YFL014W	Plasma membrane protein involved in maintaining membrane organization
		SBA1	0.695	YKL117W	Co-chaperone that binds and regulates Hsp90 family chaperones
yeast	anti-longevity	RPS30B	1	YOR182C	Protein component of the small (40S) ribosomal subunit
		TMA23	1	YMR269W	Nucleolar protein implicated in ribosome biogenesis
		URA3	1	YEL021W	Orotidine-5’-phosphate (OMP) decarboxylase
		RPS29B	0.999	YDL061C	Protein component of the small (40S) ribosomal subunit
		RLP24	0.999	YLR009W	Essential protein required for ribosomal large subunit biogenesis
		COX9	0.999	YDL067C	Subunit VIIa of cytochrome c oxidase (Complex IV)
		HOR7	0.999	YMR251W-A	Protein of unknown function
		TOM7	0.999	YNL070W	Component of the TOM (translocase of outer membrane) complex
		MFA1	0.999	YDR461W	Mating pheromone a-factor
		TAR1	0.999	YLR154W-C	Protein potentially involved in regulation of respiratory metabolism

To assess the accuracy of the predictions, we looked at the literature to see if there is experimental evidence of pro/anti-longevity effects for these genes. Based on the existing experimental evidence, we find that the model predictions are remarkably good. It turns out that—even though they are not in GenAge yet—there is experimental evidence for the pro/anti-longevity status of most of the predicted genes.

#### Predicted pro-longevity worm genes

For many of the predicted pro-longevity genes in Table 1, there already exists direct experimental evidence of pro-longevity status. Note that this evidence was not used in making the predictions, implying that the model is producing reliable out-of-sample predictions. We discuss what is known about the top 10 predicted pro-longevity genes: CLEC-196, F44E5.4, CEH-13, LPR-3, HIL-7, W04A8.4, TTH-1, GST-1, F44E5.5, and F20C5.6.

F44E5.4 and F44E5.5 encode members of the *hsp70* family of heat shock proteins. The heat shock response is well-known to have strong pro-longevity effects in *C. elegans*. Indeed, knocking in extra copies of *hsp70* extends lifespan [19] and knocking down *hsp70* via RNAi decreases lifespan and leads to rapid aging phenotypes [20]. GST-1 (Glutathione S-transferase P) is also involved in stress response—particularly, immune response—and GSTs are well-known to be pro-longevity. Overexpression (underexpression) of GSTs has been found to increase (decrease, respectively) lifespan and stress resistance [21, 22]. W04A8.4 is an uncharacterized protein that is involved in the pro-longevity effect of metformin on *C. elegans* [23]; specifically, knockdown of W04A8.4 leads to metformin resistance. This is intriguing, since metformin treatment has been shown to promote health and extend lifespan in many organisms. Homeobox protein CEH-13 exhibits pro-longevity characteristics based on experimental evidence—specifically, a *ceh-13* mutant strain has decreased lifespan compared to wildtype controls [24]. LPR-3 (LiPocalin-Related protein) is known to be involved in nematode worm locomotion, and appears to mediate the longevity-inducing effect of *daf-7* mutation [25]; additionally, expression of *lpr-3* is increased in worms fed with *rBm* *α*TX14, an *α*-neurotoxin that increases worm lifespan [26].

For the remainder of the genes in Table 1, there is suggestive experimental evidence of pro-longevity status based on associations. C-type Lectin *clec-196* expression increases and lifespan increases when *hsb-1* is knocked out [27]. Also, *clec-196* is directly adjacent to *hsp-1* on chromosome IV, suggesting possible co-involvement, and *hsp-1* (heat shock protein) is well-known to be pro-longevity. HIL-7 (Histone H1 Like) gene expression may be associated with Ethosuximide treatment, a drug that increases worm lifespan and affects DAF-16/FOXO target gene expression [28]. TTH-1 (Thymosin beta) is significantly increased in *daf-2* mutants, which are very long-lived, suggesting possible pro-longevity status by association [29]. F20C5.6 is affected by the well-known longevity genes *clk-1* and *sir-2.1*, as well as by treatment with 1-methylnicotinamide and rotenone, which are well-known for increasing worm lifespan.

This validating evidence from the literature indicates that the model predictions are surprisingly accurate. The predicted pro-longevity genes CLEC-196, HIL-7, TTH-1, and F20C5.6 are candidates for further experimental exploration.

#### Predicted anti-longevity worm genes

Similarly to the predicted pro-longevity genes, there exists experimental evidence of anti-longevity status of most of the predicted anti-longevity genes in Table 1. We discuss what is known about the top 10 predicted anti-longevity genes: MSP-59, Y59E9AR.7, RPL-39, MSP-57, MSP-81, MSP-113, MSP-19, NLP-27, and RPL-11.1.

Major sperm proteins appear to be anti-longevity based on the experimental evidence. A mutation reducing sperm production leads to significantly increased lifespan [30]. Additionally, the expression of sperm-related genes—especially major sperm protein (MSP) genes—is decreased in adult *daf-2* mutants, providing further support for an anti-longevity role of MSP genes [31].

RSP-39 and RPL-11.1 are 60S ribosomal proteins. RNAi knockdown of genes encoding ribosomal proteins consistently increases lifespan in *C. elegans*, both in the case of 40S and 60S ribosomal proteins [32]. This supports the predicted anti-longevity status.

NLP-27 (Neuropeptide-Like Protein) is the only other predicted anti-longevity gene in the top 10 list. Expression of *nlp-27*, along with other *nlp* genes, is increased in long-lived *daf-2* mutants. Further, *nlp-27* expression is reduced in a short-lived *mir-71* deletion strain. This indirect evidence by association suggests a possible pro-longevity role of NLP-27—which would contradict the predicted anti-longevity—but direct over/under-expression of *nlp-27* would be needed to establish its pro/anti-longevity status.

#### Predicted pro-longevity yeast genes

Table 1 lists the top 10 predicted pro-longevity yeast genes. Several of these predictions are borne out by direct experimental evidence via single-gene deletions—specifically, Marek & Korona [33] found that deletion of ACS1, ETR1, UBI4, and POR1 leads to decreased lifespan. Marek & Korona did not find a significant pro- or anti-longevity effect for UBC5, HSP12, or SBA1, and they do not report results for the remainder of the top 10 genes. However, UBC5 is a strong pro-longevity candidate, since it is involved in cellular stress response and mediates selective degradation of short-lived and abnormal proteins [34]. HSP12 (heat shock protein) is required for the lifespan-extending effect of dietary restriction in yeast [35], validating the pro-longevity prediction. SBA1 is also a strong pro-longevity candidate, as a chaperone-binding protein that is involved in heat shock response and is required for telomere length maintenance [36, 37]. PRE3 and PRE7 are part of the proteasome, and it is known that increased proteasome capacity extends lifespan [38], providing indirect validation of their predicted pro-longevity status. PDI1 is a downstream target of the unfolded protein response (UPR), which is well-known to be pro-longevity [39].

#### Predicted anti-longevity yeast genes

Table 1 lists the top 10 predicted anti-longevity yeast genes. As in worms, depletion of ribosomes increases lifespan [40], validating the predictions of the ribosome-biogenesis proteins RPS30B, TMA23, RPS29B, and RLP24 as anti-longevity. HOR7 is reported to influence lifespan, but the direction of the effect may be context-dependent: HOR7 deletion increases lifespan [15], whereas Schleit et al [41] find that HOR7 deletion decreases lifespan under dietary restricted conditions.

For URA3, COX9, TOM7, MFA1, and TAR1, we do not find pre-existing corroboration of the predicted anti-longevity status in the literature. TOM7 deletion has been reported to decrease chronological lifespan [42], and it does not appear to have a strong effect on replicative lifespan [33]. TOM7 is part of the translocase of the outer mitochondrial membrane (TOM) complex, and the mitochondrial membrane is well-known to be important in yeast longevity [43]. Marek & Korona [33] report a pro-longevity effect for COX9, contrary to the model prediction. (Except for COX9, the results of Marek & Korona are inconclusive for all of the genes in Table 1). Further investigation of URA3, COX9, TOM7, MFA1, and TAR1 might be interesting to pursue.

### Validation on a secondary dataset

To further evaluate the predictive accuracy of the trained pglm model, we compare the model predictions to actual lifespan measurements from a non-GenAge validation dataset. For this purpose, we use the McCormick et al [15] dataset of replicative lifespan for a comprehensive set of 4,698 single-gene deletions in yeast. Since the McCormick dataset contains lifespan measurements for deletions of many genes that do not appear in GenAge, in principle it should be well-suited as a secondary validation dataset. Using the pglm model trained on the full GenAge database for yeast with the GO+ARCHS4 feature set as predictors, we made predictions of the longevity effect of all 4,698 genes in the McCormick dataset.

**Fig 4:**
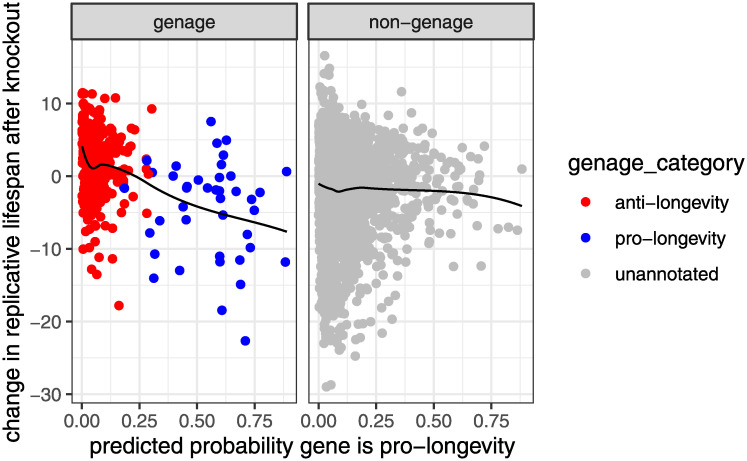
Predicted probability of a gene being pro-aging versus effect of deletion on replicative lifespan (RLS) in yeast. Probabilities are from the pglm classifier trained on the full GenAge dataset. Solid curve is a nonparametric smoother.

First, as a sanity check, we observe that among genes in GenAge, the predicted probability of a gene being pro-longevity is clearly inversely related to the change in lifespan after deletion (Fig 4, left panel). This is not surprising since it simply means that the GenAge annotations are roughly consistent with the McCormick data, and the model was able to fit the GenAge-based training data. More interestingly, we see that the model is able to predict which genes have a larger or a smaller effect on lifespan (Fig 4, left panel). For instance, among pro-longevity genes, the genes with predicted probability near 1 do indeed tend to lead to a larger decrease in lifespan. Meanwhile, among anti-longevity genes, the genes with predicted probability near 0 do indeed tend to lead to a larger increase in lifespan. Since the training data contain no information about the magnitude of the effect on lifespan, this indicates that the model is not simply recapitulating the training data, but is indeed making generalizable predictions.

Next, we compare the model predictions to the lifespan data for genes outside the GenAge database. Fig 4 (right panel) shows the change in lifespan versus the predicted probability of a gene being pro-longevity, for genes in the McCormick dataset that are not in GenAge. A downward trend in this plot would indicate concordance between model predictions and the validation data. There is an extremely slight but not convincing downward trend; thus, while suggestive, this does not provide a compelling out-of-sample validation of the model predictions. Note that the pglm classifier trained on GenAge has a strong bias toward predicting genes to be anti-longevity; see Fig 4 (right panel) and S4 Fig. This bias is due to class imbalance in the training data, since the majority of genes annotated in GenAge are anti-longevity. This is common when the training data are imbalanced, and can easily be addressed by selecting the classification threshold to yield appropriately balanced predictions.

The lack of concordance between the out-of-sample model predictions and the McCormick lifespan data may be attributable to the fact that for many genes, the McCormick data are not in agreement with the GenAge annotations of pro/anti-longevity. Specifically, many putatively pro-longevity genes led to large increases in lifespan when deleted, and many putatively anti-longevity genes led to large decreases in lifespan when deleted (Fig 4, left panel). It is not clear whether this discrepancy is primarily due to limitations of the GenAge database (e.g., bias and relatively small sample size) or limitations of the McCormick assay. Focusing on the latter possibility, recent studies have identified mechanisms by which disruption of a gene through knockout can activate compensatory mechanisms leading to a dramatically different phenotype than disruption of the same gene through knockdown, which reduces but does not eliminate expression [44]. If deletion of a single gene activates similar compensatory mechanisms in yeast, then this could explain the lack of concordance, since it would imply that the change in lifespan under a single-gene deletion is not necessarily related to that gene’s pro/anti-longevity status. A comprehensive assay of knockdowns (rather than deletions or knockouts) would shed light on this intriguing question. The discrepancy between GenAge and McCormick could also partially be due to the fact that GenAge includes results for both replicative and chronological lifespan. However, this does not fully explain the discrepancy since many of the most discordant genes were annotated as affecting replicative lifespan in GenAge.

### Functional interpretation of model predictions

To interpret the biological basis for the model predictions in terms of functional categories, for each species we retrained the pglm model on the full GenAge dataset using only GO terms as features. We extracted the 20 most influential GO terms from the trained model by ranking the regression coefficients from largest to smallest in absolute value (Table 2). Note that in this model, the coefficient is equal to the log-odds ratio (logOR) of a gene being pro-longevity when it is annotated to a GO term versus when it is not annotated to that GO term. If a GO term has a positive logOR value, then genes annotated with that GO term are more likely to be pro-longevity under the model. Conversely, a negative logOR indicates that genes annotated with that GO term are more likely to be anti-longevity.

**Table 2:** Top GO terms identified by the pglm (GLM-Net) algorithm. logOR: log-odds ratio. OR: odds ratio. Positive logOR indicates a gene annotated to that GO term is more likely to be pro-longevity. BP: biological process, CC: cellular component, MF: molecular function.

Species	ID	logOR	OR	Type	Description
worm	GO:0006412	-0.98	0.38	BP	translation
	GO:0005634	0.89	2.4	CC	nucleus
	GO:0015031	0.82	2.3	BP	protein transport
	GO:0005789	0.77	2.1	CC	endoplasmic reticulum membrane
	GO:0005840	-0.73	0.48	CC	ribosome
	GO:0009792	0.68	2	BP	embryo development ending in birth or egg hatching
	GO:0006511	0.66	1.9	BP	ubiquitin-dependent protein catabolic process
	GO:0009408	0.66	1.9	BP	response to heat
	GO:0043005	0.65	1.9	CC	neuron projection
	GO:0030150	0.6	1.8	BP	protein import into mitochondrial matrix
	GO:0055120	-0.6	0.55	CC	striated muscle dense body
	GO:0005783	0.6	1.8	CC	endoplasmic reticulum
	GO:0046872	-0.53	0.59	MF	metal ion binding
	GO:0005739	-0.52	0.59	CC	mitochondrion
	GO:0006281	-0.52	0.59	BP	DNA repair
	GO:0035556	-0.52	0.59	BP	intracellular signal transduction
	GO:0045893	0.52	1.7	BP	positive regulation of transcription; DNA-templated
	GO:0008289	0.52	1.7	MF	lipid binding
	GO:0048477	0.5	1.6	BP	oogenesis
	GO:0003824	0.49	1.6	MF	catalytic activity
yeast	GO:0001302	1.8	5.8	BP	replicative cell aging
	GO:0006915	0.87	2.4	BP	apoptotic process
	GO:0016020	-0.82	0.44	CC	membrane
	GO:0005634	0.73	2.1	CC	nucleus
	GO:0000183	0.72	2.1	BP	chromatin silencing at rDNA
	GO:0005624	0.71	2	CC	membrane fraction
	GO:0007049	0.67	1.9	BP	cell cycle
	GO:0005739	0.64	1.9	CC	mitochondrion
	GO:0005515	-0.64	0.53	MF	protein binding
	GO:0003824	0.61	1.8	MF	catalytic activity
	GO:0031307	0.56	1.7	CC	integral component of mitochondrial outer membrane
	GO:0000723	0.55	1.7	BP	telomere maintenance
	GO:0005758	0.53	1.7	CC	mitochondrial intermembrane space
	GO:0055085	0.53	1.7	BP	transmembrane transport
	GO:0017111	0.52	1.7	MF	nucleoside-triphosphatase activity
	GO:0006811	0.51	1.7	BP	ion transport
	GO:0006281	0.5	1.7	BP	DNA repair
	GO:0034599	0.48	1.6	BP	cellular response to oxidative stress
	GO:0008270	-0.48	0.62	MF	zinc ion binding
	GO:0045861	0.47	1.6	BP	negative regulation of proteolysis

#### Top GO terms for worm

The current literature supports a strong longevity effect for many of the top categories in Table 2. Translation inhibition is known to increase lifespan [32], so a large negative coefficient for the *translation* and *ribosome* GO terms makes sense. Protein homeostasis is known to be key to longevity [45], so it makes sense that the model has positive coefficients for *protein transport*, *endoplasmic reticulum membrane*, and *endoplasmic reticulum*. Ubiquitin-mediated proteolysis is known to be important for promoting longevity, implying that a positive coefficient for *ubiquitin-dependent protein catabolic process* makes sense. Heat shock response is known to extend lifespan, and indeed, the model has a positive coefficient for *response to heat*. Activation of the mitochondrial unfolded protein response is known to promote longevity [46], so a positive coefficient for *protein import into mitochondrial matrix* makes sense. Mitochondria are known to be important for longevity [47], so a large coefficient for *mitochondria* makes sense; further, inhibition of mitochondrial respiration is known to extend lifespan [48], so a negative sign for the coefficient could make sense. Similarly, the importance of *DNA repair* makes sense, and surprisingly, in some cases, DNA repair gene knockdown increases lifespan, possibly due to compensatory biological mechanisms [49]; thus, a negative coefficient is, in fact, consistent with the literature.

#### Top GO terms for yeast

For yeast, Table 2 shows the top longevity-related GO terms in the model. The importance of these terms is consistent with the current literature, but the appropriate sign of the coefficient is not always clear, since the genes annotated to each GO term may have contradictory pro/anti-longevity effects and further, there may be compensatory relationships between terms due to correlated predictors.

*Replicative cell aging*, *apoptotic process*, and *cell cycle* obviously make sense as related to yeast aging and longevity. Mitochondrial membrane maintenance is known to be important in yeast longevity [43], and other membranes (e.g., the vacuole membrane) may also be important [50]; thus, large coefficients for *mitochondrion*, *integral component of mitochondrial outer membrane*, *mitochondrial intermembrane space*, *membrane*, *membrane fraction*, and *transmembrane transport* are consistent with the literature. Depletion of ribosomes is known to increase lifespan [40], so a negative coefficient for *chromatin silencing at rDNA* is appropriate. Telomeres are known to be important in yeast longevity [51, 52], so a large coefficient for *telomere maintenance* makes sense. Longevity effects of *cellular response to oxidative stress* are corroborated in the literature [53]. Finally, a negative coefficient for *zinc ion binding* is consistent with experimental evidence that zinc limitation extends chronological lifespan [54].

### Pathway enrichment analysis of model predictions

To further interpret the model predictions in terms of known biology, we performed pathway enrichment analysis. First, we took the list of non-GenAge genes that were predicted to be pro-longevity and tested for enrichment of KEGG pathways using the Database for Annotation, Visualization and Integrated Discovery (DAVID) v6.8 [55, 56]. Adjusting for multiple testing using the Benjamini–Hochberg correction, we found that the “Proteasome” pathway was significantly enriched (corrected p-value 0.0031). The KEGG pathway diagram in S5 Fig (used with permission from Kanehisa Laboratories [57]) indicates that several of the predicted pro-longevity genes are in the 20S proteasome core particle, particularly in *β* subunits. This is intriguing, since the proteasome is a protein complex that breaks down unneeded or damaged proteins by proteolysis, and the *β* subunits play a central role in this process [58]. Sustained proteasome activity appears to be associated with longevity based on studies of long-lived humans and rodents, and directly elevating proteasome activity increases longevity in yeast [38].

We performed the same enrichment analysis using the top predicted anti-longevity genes for yeast, and separately, the pro- and anti-longevity genes for worm. In each case, we capped the number of genes at 100. S6 Table shows the top KEGG pathway hit in each case. Notably, in both yeast and worm, the “Ribosome” pathway was highly significantly enriched with predicted anti-longevity genes (corrected p-values 7.1 × 10^−15^ and 8.1 × 10^−27^, respectively). These results are consistent with known aging biology, and since these genes are not currently in GenAge, the model predictions may offer new avenues of research.

## Discussion

### Limitations

A limitation of our models is that the pro/anti-longevity status of a gene is predicted based on how similar its GO terms and/or gene expression pattern are to genes with known pro- or anti-longevity status. This similarity does not necessarily imply that manipulation of these genes will have the predicted effect on lifespan, and further, the predictions are limited by the accuracy of the input data. This is illustrated by SIR2 and DNL4, the top two hits in S4 Table for yeast when using the GO-only model. Both SIR2 and DNL4 are annotated with the “replicative cell aging” GO term, which is strongly indicative of pro-longevity status in this model, as indicated by the odds ratio of 5.8 in Table 2. Experimental evidence is consistent with the SIR2 prediction, but not the DNL4 prediction [59]. This appears to be due to the interesting fact that although DNL4 is required for DNA repair by nonhomologous end joining (NHEJ), apparently NHEJ does not affect replicative aging in yeast [59]. Thus, in the case of DNL4, the discrepancy between prediction and experiment may be viewed as an inadequacy of this particular GO term annotation.

Another limitation is that although *S. cerevisiae* (yeast) can be haploid or diploid, our models are not ploidy-specific since much of the data we use (GenAge, GO terms, and gene expression) are not annotated in a way that indicates whether they pertain to haploid or diploid. Significant differences have been observed between haploid and diploid yeast aging [59, 60], making it difficult to know whether results for one would extend to the other. That said, overall we would expect the set of genes that are strongly involved in longevity to be similar for haploid and diploid, although the magnitude (and possibly the direction) of the pro/anti-longevity effect may vary.

Similarly, our models do not distinguish between chronological lifespan and replicative lifespan in yeast. In future work, it would be interesting to analyze chronological lifespan separately from replicative lifespan since there may be major differences.

By a fortunate coincidence, the best performing algorithm, pglm (GLM-Net), enabled us to perform functional interpretation of the results by simply considering the largest regression coefficients. In future studies on alternative datasets, higher predictive performance might be obtained with other algorithms lacking easily understandable coefficients. Under that scenario, we would recommend researchers consider alternative feature importance metrics such as those provided by the caret R package [61].

### Conclusions and future directions

We systematically compared the performance of popular machine learning algorithms in classifying genes as pro- or anti-longevity using the GenAge database and combinations of gene expression and gene ontology (GO) feature sets. We identified elastic net penalized logistic regression (pglm) as the most effective classifier and made predictions for unannotated genes. We offer our predictive probability scores as one possible tool to prioritize future experimental studies which can validate individual genes as pro-longevity mechanistically. Our approach of combining feature sets to improve predictive performance is generalizable in principle to a wider variety of model organisms as more annotations and datasets become available over time.

We encourage other computational researchers to use metrics such as area under receiver-operator curve (AUC) on held-out data from standard databases such as GenAge to assess classification performance and facilitate comparisons across studies. We suggest that future comprehensive longevity assays consider using knockdowns instead of deletions and knockouts, due to the existence of compensatory mechanisms that are known to mitigate the effects of knockouts [44]; this may improve the concordance between predictions and experimental evidence. Additionally, there appears to be a need for increased focus on pro-longevity genes as opposed to anti-longevity genes, since pro-longevity genes are much less common in the GenAge database.

In addition to genetic variation, environmental factors such as exposure to drugs or other chemical compounds are known to influence longevity [4, 62, 63]. Future studies may benefit from our computational framework in this context, for example, by using outcome variables from the DrugAge database [64] to train classifiers or regression models. However, a key challenge will be to identify suitable covariates analogous to gene expression or GO terms. One intriguing possibility would be to convert the molecular structure of each drug into a vector of continuous features [65].

Finally, it is clear that genes act in networks rather than individually—for instance, top-down analysis has identified the nutrient sensing pathway, the mitochondrial effector pathway, and the proteostasis pathway as collectively regulating single-cell longevity [66]. Thus, network-based approaches are likely to yield further insights into the molecular mechanisms of aging. In particular, while we have considered only single-gene manipulations, it would be valuable to be able to predict the effect of multiple simultaneous interventions. This is very challenging in general, but it might be possible to exploit special structure in the mechanisms of aging—for instance, recent papers have argued that aging may be governed by a single global state variable, based on the finding that many diverse interventions lead to a temporal scaling of survival curves in *C. elegans* and *S. cerevisiae* [67, 68].

## Methods

### Acquisition and preprocessing of datasets

Binary pro/anti-longevity annotations were accessed from the GenAge model organisms database build 19 [6], available at http://genomics.senescence.info/genes. We used the subset of genes for yeast and worm, and we excluded ambiguous annotations (e.g., if GenAge lists two studies for a gene, one finding it to be pro-longevity and the other finding it to be anti-longevity). GO annotations for all genes were downloaded from the BioMart ENSEMBL database (release 93, July 2018) using the biomaRt package in Bioconductor (version 3.7). For both species, gene expression data in the form of RNA-Seq read counts were obtained from the ARCHS4 database version 1 [16], currently available at https://amp.pharm.mssm.edu/archs4/archs4zoo.html. For yeast only, we acquired the Deleteome gene expression microarray dataset [17], currently available at http://deleteome.holstegelab.nl (no version available but last updated May 2014). For worm only, we obtained gene expression data from the single-cell RNA-Seq Worm Cell Atlas [18], currently available at http://atlas.gs.washington.edu/worm-rna (no version available but last updated August 2017). We reduced the dimensionality of the Worm Cell Atlas data by summing the unique molecular identifier (UMI) counts across all cells within the same tissue, so that each feature is a “pseudobulk” tissue rather than a single cell.

Replicative lifespans (RLS) for 4,698 single-gene deletion yeast strains were obtained from McCormick et al [15] in June 2017. Perturbation genotypes with percent_change greater than 30 and set_lifespan_count less than or equal to 5 were excluded based on the authors’ recommendations. We merged results for the same genotype across replicate experiments in the following way. The outcome for each genotype in a single replicate was quantified as the mean of RLS in the perturbation group minus the mean of RLS in the control group. To obtain a single value for the genotype across all replicates, we then computed a weighted average of the outcome values from each replicate, where the weights corresponded to the sample sizes in each group. This ensured that replicates with more observations contributed more to the final value. We refer to this as the McCormick dataset.

### Data normalization and quality control

All gene expression measurements were normalized to account for sample-specific biases. Specifically, the Deleteome data were already normalized, the ARCHS4 read counts were converted to transcripts-per-million (TPM), and the Worm Cell Atlas UMIs were converted to counts-per-million (CPM). The normalized counts were then log transformed with a pseudocount of one. For Deleteome, genes that were variable in controls and non-responsive mutants were excluded, since these data were likely to contain mostly noise. For each species, we used the subset of genes with no missing values across all feature types (GO features and the two sources of gene expression features), resulting in 703 worm genes (246 pro-longevity, 457 anti-longevity) and 368 yeast genes (46 pro-longevity, 322 anti-longevity). Features with no variation across the included genes were discarded. For yeast, the number of retained features was 3268, 700, and 1390 for ARCHS4, Deleteome, and GO terms, respectively. For worms, the number of features was 2935, 270, and 2051 for ARCHS4, Worm Cell Atlas, and GO terms, respectively. All gene expression features were centered and scaled to have mean zero and standard deviation 0.5 as suggested by [69], while binary features (GO) were not centered and scaled. The five sets of features considered for each species were (1) ARCHS4 alone, (2) GO alone, (3) GXP alone (Deleteome for yeast, Worm Cell Atlas for worm), (4) GO combined with ARCHS4, and (5) GO combined with GXP.

### Comparison of predictive performance by algorithm and feature set

To assess predictive performance of different combinations of feature sets, each dataset (consisting of the binary GenAge outcome for a single species matched with one of the five feature sets) was split into 5 external cross-validation (CV) folds. Within each fold, machine learning classifiers were fit to the training data using the caret package version 6.0 [61] in the R programming environment (version 3.5). The same partitioning of the data was preserved across algorithm runs to ensure identical training and test conditions. The algorithms used were k-nearest neighbors (knn, R package kknn version 1.3.1), naive Bayes (nb, R package naivebayes forked at version 0.9.2 and modified for numerical stability, https://github.com/willtownes/naivebayes), gradient boosted trees (xgb, R package xgboost version 0.8.0), support vector machine with radial basis function (svm, R package kernlab version 0.9), and logistic regression with elastic net penalty (pglm, R package glmnet version 2.0). Hyperparameters (S1 Table) were selected by grid search using repeated 10-fold internal CV with two repeats within each training fold using the Kappa criterion. Note that this means each algorithm could potentially use different hyperparameter values across the five external CV folds. For all algorithms except naive Bayes, the grid consisted of default caret values. For naive Bayes, the Laplace correction was set to zero, kernel smoothing was always used, and the adjustment to the probabilities was chosen between 0.5 and 1.0. Additionally, for naive Bayes only, many features with near-zero variance caused numerical instabilities and were excluded. Having chosen a final set of hyperparameters for each training fold, the predicted probabilities were computed for the held-out test data and the area under the receiver-operator curve (AUC) was computed to quantify prediction performance (discrimination). An AUC value of 1 indicates perfect classification performance, whereas an AUC of 0.5 signifies performance no better than random, or simply always predicting the majority class.

### Model fitting for novel predictions and validation

For the results in sections ‘Novel predictions of pro/anti-longevity genes’ and ‘Validation on a secondary dataset’, the best-performing algorithm (pglm) was retrained on all of the GenAge data for each species with the combined GO plus ARCHS4 feature set. The hyperparameter grid was expanded to 21 alpha values (evenly spaced between zero and one, inclusive), and 97 automatically selected lambda values using five-fold CV. For worm, the optimal alpha was 0.05 (close to an L2 ridge penalty). For yeast, the optimal alpha was 0.5 (an even mix between ridge and the L1 lasso penalty). Using the optimal hyperparameters, predictive probabilities were computed for all genes.

### Model fitting for functional interpretations

For the results in section ‘Functional interpretation of model predictions’, for each species the pglm algorithm was retrained on the full GenAge dataset using GO features only. This choice of feature set was used to enable interpretation of regression coefficients. Here, the hyperparameter grid was the same 21 alpha values and 97 automatically selected lambda values with five-fold CV. The optimal alpha values were 0.15 for worm and 0.10 for yeast (both closer to ridge than lasso).

## Supporting information

S1 Fig Comparison of predictive performance of machine learning algorithms on classifying genes as pro- or anti-longevity. pglm: elastic net penalized logistic regression, svm: support vector machine with radial basis function, xgb: gradient boosted trees, nb: naive Bayes, knn: k-nearest neighbors, gxp: gene expression, ROC: receiver-operator curve.
(EPS)

S2 Fig Comparison of predictive performance of different feature sets on classifying genes as pro- or anti-longevity. pglm: elastic net penalized logistic regression, svm: support vector machine with radial basis function, xgb: gradient boosted trees, nb: naive Bayes, knn: k-nearest neighbors, gxp: gene expression, ROC: receiver-operator curve.
(EPS)

S3 Fig Predictive probabilities of genes being pro-longevity under GO+ARCHS4 feature set versus GO only feature set. (EPS)

S4 Fig Distribution of predictive probabilities after training elastic net penalized logistic regression (pglm) on the full GenAge dataset with GO terms and ARCHS4 gene expression as features. (EPS)

S5 Fig Proteasome KEGG pathway schematic [57]. Subunits containing predicted pro-longevity genes are indicated with red stars. (Copyright of Kanehisa Laboratories, used with permission.).
(TIFF)

S1 Table Hyperparameters used. pglm: elastic net penalized logistic regression, svm: support vector machine with radial basis function, xgb: gradient boosted trees, nb: naive Bayes, knn: k-nearest neighbors.
(CSV)

S2 Table Top worm pro-longevity genes not in GenAge predicted using only GO terms as features. (CSV)

S3 Table Top worm anti-longevity genes not in GenAge predicted using only GO terms as features. (CSV)

S4 Table Top yeast pro-longevity genes not in GenAge predicted using only GO terms as features. (CSV)

S5 Table Top yeast anti-longevity genes not in GenAge predicted using only GO terms as features. (CSV)

S6 Table Top KEGG pathway hits from DAVID enrichment analysis. (CSV)

S1 Data All genes pglm predictions for yeast with GO+ARCHS4 features. (XLSX)

S2 Data All genes pglm predictions for worm with GO+ARCHS4 features. (XLSX)
