# Supplementary figures and images for: Identifying longevity associated genes by integrating gene expression and curated annotations
Source: PLoS Comput Biol. 2020 Nov 30;16(11):e1008429. doi: 10.1371/journal.pcbi.1008429 (PMC7728194; doi:10.1371/journal.pcbi.1008429)

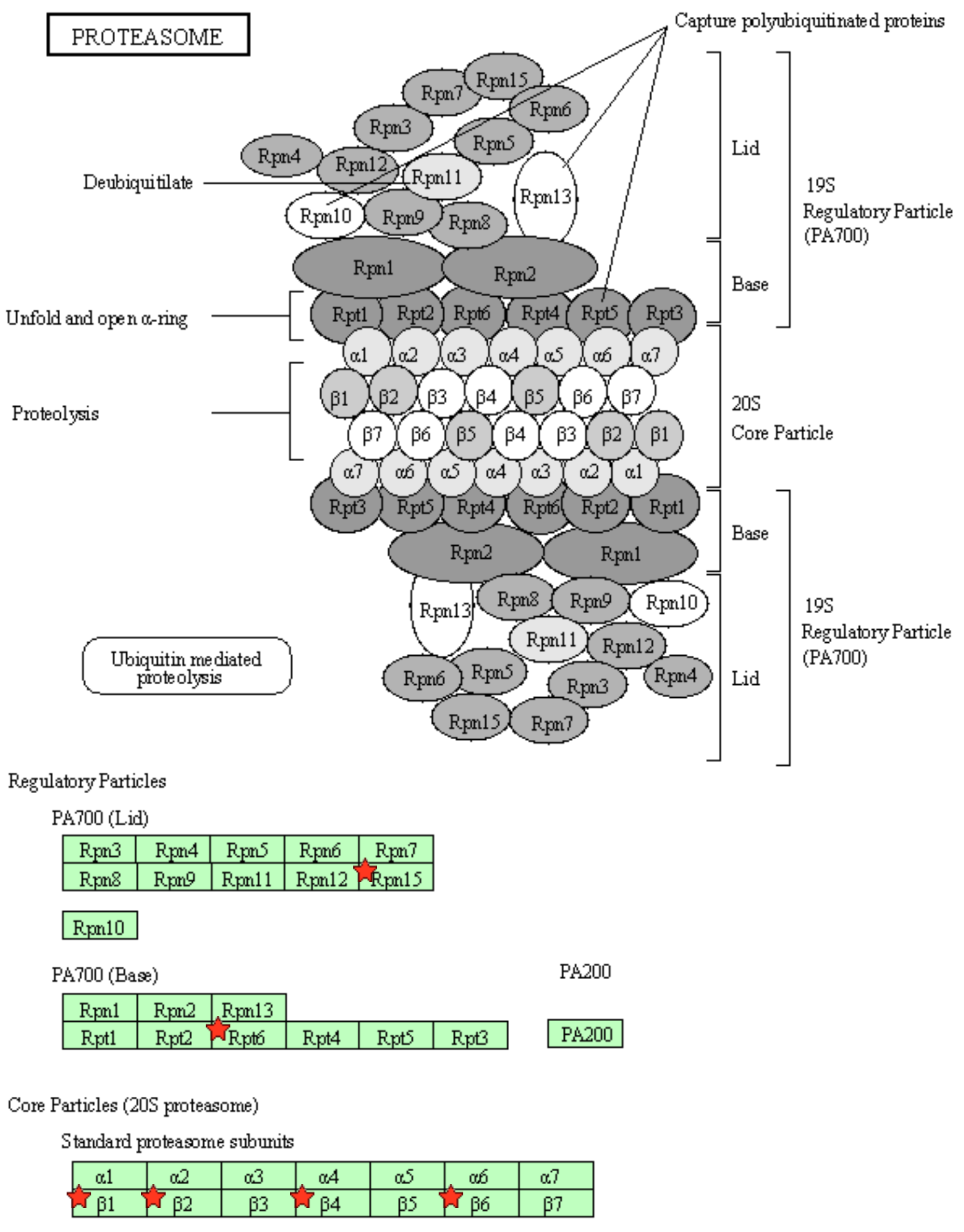

Supplement: S5 Fig — Subunits containing predicted pro-longevity genes are indicated with red stars. (Copyright of Kanehisa Laboratories, used with permission.). (TIFF) [file pcbi.1008429.s005.tiff]
